# Monoclonal Culture and Characterization of Symbiodiniaceae C1 Strain From the Scleractinian Coral *Galaxea fascicularis*

**DOI:** 10.3389/fphys.2020.621111

**Published:** 2021-01-18

**Authors:** Jun Wang, Jiaqi Chen, Shaoyu Wang, Fuyu Li, Chengchong Fu, Yan Wang

**Affiliations:** State Key Laboratory of Marine Resource Utilization in South China Sea, College of Marine Sciences, Hainan University, Haikou, China

**Keywords:** *Cladocopium* sp. C1, monoclonal strain, *Galaxea fascicularis*, ultrastructure, stereology, growth rate

## Abstract

The symbiosis between cnidarian hosts and photosynthetic dinoflagellates of the family Symbiodiniaceae (i.e., zooxanthellae) provides the energy foundation of coral reef ecosystems in oligotrophic waters. The structure of symbiont biota and the dominant species of algal symbiont partly shape the environmental adaptability of coral symbiotes. In this study, the algal symbiont cells were isolated from the tentacles of *Galaxea fascicularis*, a hermatypic coral with obvious differentiation in heat resistance, and were cultured *in vitro* with an improved L1 medium. An algal monoclonal cell line was established using separated algal culture drops and soft agar plating method, and named by GF19C1 as it was identified as *Cladocopium* sp. C1 (Symbiodiniaceae) based on its ITS1, ITS2, and the non-coding region of the plastid psbA minicircle (*psbA*^*ncr*^) sequences. Most GF19C1 cells were at the coccoid stage of the gymnodinioid, their markedly thickened (ca. two times) cell wall suggests that they developed into vegetative cysts and have sexual and asexual reproductive potential. The average diameter of GF19C1 cells decreased significantly, probably due to the increasing mitotic rate. The chloroplasts volume density of GF19C1 was significantly lower than that of their symbiotic congeners, while the surface area density of thylakoids relative to volumes of chloroplasts was not significantly changed. The volume fraction of vacuoles increased by nearly fivefold, but there was no significant change in mitochondria and accumulation bodies. Light-temperature orthogonal experiments showed that, GF19C1 growth preferred the temperature 25 ± 1°C (at which it is maintained post-isolation) rather than 28 ± 1°C under the light intensity of 42 ± 2 or 62 ± 2 μmol photons m^–2^ s^–1^, indicating an inertia for temperature adaptation. The optimum salinity for GF19C1 growth ranged between 28–32 ppt. The monoclonal culture techniques established in this study were critical to clarify the physiological and ecological characteristics of various algal symbiont species, and will be instrumental to further reveal the roles of algal symbionts in the adaptive differentiation of coral-zooxanthellae holobionts in future studies.

## Introduction

The establishment of specialized intracellular symbiotic relationship between reef-building corals and photosynthetic dinoflagellates (Symbiodiniaceae, also known as zooxanthellae) is the primary energy source for reef ecosystems to flourish in oligotrophic tropical shallow waters. Photosynthesis of symbiotic algae can supply more than 95% of the nutritional needs of the corals and contribute to the calcification of reef corals to form the carbonate framework of coral reefs (Mallela, 2013). However, due to the essential differences in metabolic rates and nutritional requirements between corals and algal symbionts, the precise homeostasis necessary to maintain the symbiotic relationship is highly sensitive to environmental stresses (Obura, 2009), especially to the synergistic stress of light intensity and temperature variations (Lesser and Farrell, 2004; Ferrier-Pages et al., 2007; Hawkins et al., 2015). A thermal perturbation as little as 1°C above the average summer maxima could cause the breakdown of this symbiosis and lead to coral bleaching (Hume et al., 2015). Since 1980s, worldwide coral bleaching events caused by global warming have become more and more frequent, with too short intervals allowing for a full recovery of mature assemblages. As a result, mass coral mortality and the severe degradation of the coral reefs structure and ecological functions occurred (Hoegh-Guldberg et al., 2007; Hughes et al., 2017). Even more worrying, as global warming in progression, local extreme weather conditions are more frequently seen, and the coral reef ecosystems are likely to further decline (Hughes et al., 2018). Therefore, analyzing the adaptation and resilience of reef-building coral holobionts has become the focus of coral reef protection and resilience. This is bound to start with the two symbiotic parties, respectively, to clarify the physiological and ecological characteristics and environmental adaptation potentials of reef-building corals (Shinzato et al., 2011; Ying et al., 2018) and symbiotic algae (Lin et al., 2015; Aranda et al., 2016). And then, bring it to the level of holobiont as a unique biological entity of evolutionary selection for integrated research (Rosenberg, 2013).

The algal symbiont used to belong to *Symbiodinium*, a genus with obscure morphological and taxonomic characteristics with complex phylogenetic lineages (LaJeunesse et al., 2018). The species of this genus formed a complex symbiotic relationship with numerous categories of marine invertebrates (Trench, 1993). In the past 30 years, DNA sequences and molecular biology techniques have been used to establish a comprehensive phylogenetic relationship for algal symbionts derived from various marine invertebrates. Based on nuclear 18S-rDNA and restriction fragment length polymorphisms (RFLPs) (Rowan and Powers, 1991a,b), chloroplast 23S-rDNA gene sequence (Santos et al., 2002; Pochon et al., 2006; Pochon and Gates, 2010), *Symbiodinium* was classified into nine (A–I) genetically distinctive clades. And each clade is further divided into multiple subclades based on the nuclear internal transcribed spacer (ITS) regions (LaJeunesse, 2001; Van Oppen et al., 2005). Recently, LaJeunesse et al. (2018) systematically revised the evolutionarily divergent *Symbiodinium* Clade A–G to seven genera in the family Symbiodiniaceae, and some subclades or genetic strains are described as species within those genera. With increasing phylogenetic, ecological, and biogeographic evidences available, more novel genera and species will be likely uncovered and classified in the family Symbiodiniaceae (LaJeunesse et al., 2018). The host species associated with algal symbionts are highly diverse, even when spoken of reef-building coral hosts, they are also miscellaneous. Since the existence of symbiont-host specificity at the species level (Trench, 1993), the lineages of genetic differentiation of symbiotic algae, combining with that of coral hosts, have indicated much greater genetic and functional diversities in the algal symbionts of reef-building corals (Barshis et al., 2014). Certainly, the establishment of *in vitro* monoclonal culture of host-associated algal symbiont strains would be necessary to elucidate the species identification and characterization through collecting and analyzing their physiological and ecological data. In addition, such efforts could facilitate to reveal the roles of algal symbionts in building, maintaining, breaking down and reconstruction of the symbiosis. Nevertheless, due to the numerous difficulties in the establishment of *in vitro* culture strains (Schoenberg and Trench, 1980a), the studies on the host-associated monoclonal algal symbiont cultures are still limited (Chakravarti and Van Oppen, 2018).

*Galaxea fascicularis*, a massive coral with large polyps, is mainly distributed in the tropical and subtropical coral reef areas of the Indian- Pacific Ocean (Veron, 2000). It is also the dominant species on the fringing reefs of Hainan Island (Chen et al., 2013; Wang et al., 2013). *G. fascicularis* species includes two morphologically and genetically differentiated lineages characterized by the microbasic p-mastigophores (MpM) types of tentacular nematocyst (Hidaka, 1992) and mitochondrial genotypes (Watanabe et al., 2005) around Hainan Island (Wu, 2018; Wepfer et al., 2020). These two lineages demonstrate significant differences in heat resistance (Xu, 2019), indicating the potential of *G. fascicularis* as an ideal model for exploring the genetic basis of heat resistance differentiation of corals. Thus, the establishment of *in vitro* monoclonal cultures of *G. fascicularis* associated algal symbiont species, followed by characterization of their morphological, physiological and ecological traits, would be necessary and helpful to reveal the symbiosis flexibility behind the differentiations in environmental adaptability between the two lineages, as well as the interaction between corals and symbionts within the holobiont. In this study, we conceived and developed the techniques for successful isolation and cultivation of the monoclonal symbiotic algal strain of *G. fascicularis*. and then characterized the first monoclonal algal strain GF19C1 (Supplementary Figure 1).

## Materials and Methods

### Coral Samples and Algal Symbiont Identification

The scleractinian coral *Galaxea fascicularis* (GF) samples (one piece/colony, 23 pieces in total) were collected from West Island (18°14″16″ N, 109°21″54″ E, Sanya, Hainan, China) in April, 2018. The sample collection was approved and assisted by the Management Office of Sanya National Coral Reef Nature Reserve. Two polyps were taken down from each coral piece, and fixed in triplicate 95% alcohol. The living samples were brought back to the laboratory and maintained in the aquaria at the College of Marine Science, Hainan University under the following conditions: seawater renewal rate at 1,000 L h^–1^; temperature at 26 ± 1°C; salinity of 32–34 ppt; light intensity of 160 μmol photons m^–2^ s^–1^ and photoperiod at 12 h light: 12 h dark.

The GF symbiote DNA were extracted using modified CTAB method (Mieog et al., 2009). In brief, freshly fixed tissue filaments were cut into pieces and suspended in 800 μL CTAB extract (2% CTAB, 1.4 M NaCl, 20 mM EDTA, 100 mM Tris–HCl, 20 μg/mL proteinase K, pH 8). After adding 3–5 glass beads (diameter 3 mm) in the tube, the tissue pieces were grinded for 5 min in Tissuelyser-48 grinder (Shanghai JingXin Industrial Development Co., Ltd.), and then incubated overnight at 60°C. The total DNA was extracted with 800 μL chloroform/isopentanol (24:1), and precipitated with isopropanol at −20°C. The DNA pellets was washed by 70% ethanol and air-dried, followed by suspended at 200 μL 0.01M TE (pH 8). Subsequently, the algal symbiont nuclear small subunit (n18S)-rDNA was amplified by PCR using the primers ss5 and ss3z (Rowan and Powers, 1991b). PCR solution (10 μL) contained 1 × Taq-HS PCR Master Mix [Mona (Wuhan) Biotechnology Co., Ltd.], ∼20 ng template DNA, 5 pmol of each primer. Cycling profiles were 94°C for 5 min followed by 30 cycles of 94°C for 1 min, annealing at 55°C for 2 min, extension at 72°C for 3 min, and a final extension at 72°C for 10 min. The PCR products were digested with *Taq*I restriction enzyme to generate RFLPs (restriction fragment length polymorphism) and visualized by electrophoresis separated on 2.5% agarose gels. The clade (genus) was identified according to the RFLP patterns (Santos et al., 2002). Accordingly, the coral individual 5gw14, associated with clade C symbiont, were selected for the following isolation of algal symbiont.

### Isolation and Monoclonal Culture of Algal Symbionts

The ordinary tentacles were sampled with sterilized tweezers, and were rinsed with sterilized sea water to remove the broken septa (Supplementary Figure 2A). Each tentacle was put in an eppendorf tube, and then break the tentacle with pipette tip to release the symbiotic algae. Tissue fragments were precipitated by inching centrifugation, and the suspending algal cells were transferred into a new tube (Supplementary Figure 2B, 1st tube) containing the modified L1 medium (Z1 medium, Supplementary Table 1). In suspensions, adjust the cell density to 500 cells/mL, then sample several 5 μL droplets onto microscopy slides and observe cells under a microscopy. Transfer 4–5 algal cells into fresh tubes (2nd, 3rd, and 4th tube, Supplementary Figure 2B), and then culture them with Z1 medium. In 6 weeks or so, adjust the cell density to 2,000 cells/mL and spread 50 μL algal liquid on a soft agar plate containing antibiotics to obtain clonal axenic culture, until the microscopic confirmation of the absence of contamination and monoclonal algal colonies appeared on the plate (ca. 10–12 weeks later, Supplementary Figure 2C). The well-developed, clearly isolated and dense colonies were then transferred into Z1 liquid medium for extended cultivation. Finally, the axenic algal cells were inoculated into fresh Z1 medium every 2 weeks and cultured continuously (Supplementary Figure 2D) under the following conditions: temperature at 25 ± 1°C, light intensity of 45 ± 5 μmol photons m^–2^ s^–1^, photoperiod at 14 h light: 10 h dark. The soft agar plate medium contains: 1.5 × Z1, plus 75 mg/L NaNO_3_, 35 mg/L NH_4_Cl, penicillin (final concentration 200 μg mL^–1^), streptomycin 100 μg mL^–1^, kanamycin 100 μg mL^–1^ (Lin et al., 2015), and 0.5% (w/v) agar powder (Becton, Dickinson and Company, United States).

### Identification of the Monoclonal Algal Strains

The genomic DNA of *in vitro* cultured algae was extracted as mentioned above. The ITS1 and ITS2 regions were amplified by primer symITS1 (Van Oppen et al., 2001), ITSintfor2 (LaJeunesse and Trench, 2000) and ITS-Reverse (Coleman et al., 1994). PCR was conducted in a 35 μL reaction solution containing ca. 50 ng template DNA, and 1 × MonAmp HS Taq Mix [Mona (Wuhan) Biotechnology Co., Ltd.], 17.5 pmol of each primer. The PCR profile was: 94°C for 5 min followed by 40 cycles of 94°C for 30 s, annealing for 30 s at 59°C (for ITS1) or 51°C (for ITS2), extension at 72°C for 30 s, and a final extension at 72°C for 5 min. For further confirming the phylogenetic identity of the algal strain, sequences of the non-coding region of the plastid psbA minicircle (*psbA*^*ncr*^) were amplified using primers 7.4-Forw and 7.8-Rev (Moore et al., 2003). The PCR conditions are as followed as: 94°C for 2 min; then 40 cycles at 94°C 10 s, 55°C for 30 s, and 72°C for 2 min; followed by a final extension at 72°C for 10 min (LaJeunesse and Thornhill, 2011). The purified PCR amplicons were directly (Sanger) sequenced in both directions (ABI 3730XL DNA Sequencer) by Tsingke Company (Guangzhou, China). Mega X (Kumar et al., 2018) were used to align those sequences (Supplementary Material 1) with the associated homologous sequences of ITS1 (AF380530-AF380565, Van Oppen et al., 2001), ITS2 (GU111863-GU111905, LaJeunesse et al., 2010) and *psbA*^*ncr*^ (JQ043677-JQ043719, LaJeunesse and Thornhill, 2011), and then Neighbor-Joining phylogeny trees were constructed (Supplementary Material 1).

### Ultrastructure and Stereometry of the Monoclonal Algal Cells

The *in vitro* algal cells were collected from 2 mL culture by centrifugation and then fixed using 1 mL 2.5% (v/v) glutaraldehyde. For the *in situ* symbiont control, a few inner tissues from a polyp of 5gw14 were sampled and fixed with 1 mL 2.5% (v/v) glutaraldehyde. Upon fixation at room temperature for 2 h, the tissues or algae were embedded in 1% (w/v) agarose, and rinsed with 0.1 M phosphate buffer (pH 7.4). The agar blocks were post-fixed in 1% osmium tetroxide for 2 h at room temperature followed by rinsing with 0.1 M phosphate buffer (pH 7.4), and then dehydrated sequentially with 50–100% gradient alcohol, replaced with 100% acetone, and embedded in Epon 812. Leica EM UC7 ultramicrotome was used to make the thin sections (60–80 nm), which were stained with 2% (w/v) saturated uranium acetate alcohol solution and lead citrate. Images of *in situ* control and *in vitro* cultured algal cells were processed under a HT7700 transmission electron microscopy (Hitachi, Japan).

Randomly selected ten section micrographs showing typical features of algal organelles from both cultured and tissue sections (15–20 K final magnification) were used to assess the volume fractions of chloroplasts, mitochondria, accumulations and vacuoles by Adobe Photoshop CC software based on principles of stereology (Elias and Hyde, 1980). The ratio of chloroplast/mitochondrion/accumulation/vacuole to total cell area of each cell was calculated by the respective corresponding numbers of pixels. In order to avoid the effect of differential image resolutions, we used the image scale tool to measure the central width of three random chloroplasts in each cell and counted the number of thylakoid lamellae. The mean number of thylakoid lamellae per unit width represents the surface density of thylakoid lamellae relative to chloroplast volume (SDTL) (Lesser and Shick, 1990). Similarly, the mean thickness of cell wall from those 10 algal cells of each group was determined. For each cell, three clear cell wall locations were randomly measured.

To analyze the volume changes of *in vitro* cultured algae, the diameters of 50 algal cells (both *in vitro* and *in situ* from tentacles of the coral 5gw14) were measured by using objective micrometer (40×) under a light microscope, respectively. Meanwhile, the cell division ratio of the two groups were also calculated. Total of randomly selected 50 cells were counted each time, and the mean measure was obtained by three counts. The criteria for cells in division was based on the evidence that there is a distinct cleavage furrow in the center, and the two dividing cells are wrapped in one maternal wall.

### Orthogonal Experimental Analysis of Suitable Light and Temperature

Since isolation, the algae have been cultured under the conditions of 25 ± 1°C and 42 ± 2 μmol photons m^–2^ s^–1^. Preliminary experiments showed they are sensitive to the increase of temperature and light intensity and the growth rate is low during 8 months after isolation. Therefore, when testing the suitable photo-temperature conditions for its growth with orthogonal experiments, the parameters were set slightly higher: temperature (T1: 25 ± 1°C, T2: 28 ± 1°C), light intensity (L1: 42 ± 2, L2: 62 ± 2 μmol photons m^–2^ s^–1^). Other conditions are as follows: salinity 28 ppt, pH 8.05, and 12 h photoperiod, batch culture in small chambers. Each set of treatments has three replicates (*n* = 3). The algal cell density and growth rate were measured in 7 and 14 days, respectively. The specific growth rate (*K*, doubling/day) was calculated as:

$$K=\left(\text{ln}\,Z_{\text{t}}-\text{ln}\,Z_{0}\right)/t$$

Where *Z*_0_ is the cell density at the starting point of the experiment, and *Z*_*t*_ is the cell density at day 7 and 14, respectively, *t* is the duration of cultures in days.

### Test of Optimum Salinity for Growth

The test was performed at 12 months after isolation. Considering that the algae were transferred from coral cells to the external environment, they may be more sensitive to salinity changes. Therefore, a dense salinity gradient was set to determine the optimum salinity: 10, 15, 20, 24, 26, 28, 30, 32, 35, and 40 ppt. Each salinity has three culture replicates (*n* = 3). The salinity gradients of cultures were obtained by diluting 0.2 μm filtered seawater (FSW) with distilled water or amended with NaCl (pH = 8.05). According to the results of orthogonal experiment and preliminary experiments, the temperature was set at 25 ± 1°C and light condition was under 62 ± 2 μmol photons m^–2^ s^–1^ with 12 h photoperiod. Batch culture in small chambers. The algal cell densities and growth rates were measured on days 7, 21, and 28, respectively, as above-mentioned.

### Statistical Analyses

Statistical analysis was carried out with IBM SPSS Statistics 26.0. Kruskal–Wallis Test was used to evaluate the significance of differences in morphological and structural parameters between GF19C1 and its symbiotic congeners (SC). One way ANOVA was used to investigate the salinity (ten levels) on the growth rate of GF19C1, with Tukey *post hoc* comparisons to locate significant differences. Similar analytical methods are used to compare the effects of temperature (two levels) and illumination (two levels) on the algal growth rates. The growth rate data of 7 days in the orthogonal experiment of light and temperature were log-transformed before analysis, because they did not conform to the homogeneity of variance. Differences with *P* < 0.05 were considered as significant for all analysis.

## Results

### Identification of the Algal Symbionts of *Galaxea fascicularis*

**FIGURE 1:**
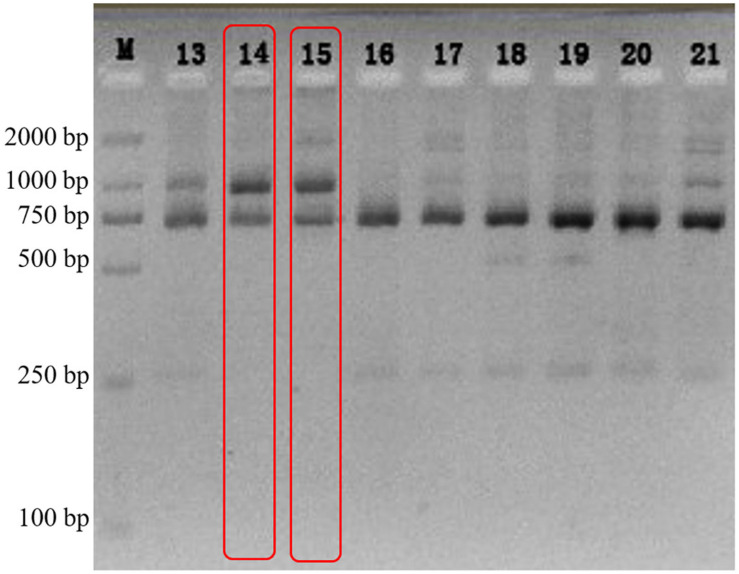
The electrophoretogram of n18S-rDNA *Taq*I-generated RFLPs from *G. fascicularis* DNA.

The electrophoretogram of n18S-rDNA *Taq*I-generated RFLPs from a *Galaxea fascicularis* population (*n* = 23) showed that, only the fingerprint patterns of individuals 5gw14 and 5gw15 were identical to that of type C coral (Santos et al., 2002; two bands, 700 bp and 1,000 bp as shown in Figure 1), and 18 individuals were associated simultaneously with type C and D, while the other three samples were associated with type D only. The individual 5gw14 was then chosen to isolate the algal symbionts.

### Phylogenetic Identity of the Monoclonal Algal Strains

The internal transcribed spacer (ITS1 and ITS2) sequences obtained from monoclonal cultured algae were all identical to *Cladocopium* sp. C1 ITS1 (AF380551, Van Oppen et al., 2001) and *Cladocopium* sp. C1 ITS2 (GU111864, LaJeunesse et al., 2010), respectively. The algal *psbA*^*ncr*^ sequence is identical to *Cladocopium* sp. C1 isolate 152 clone 16 *psbA*^*ncr*^ (JQ043704), which differs from *Cladocopium goreaui* (JQ043677) by one base transversion and four insertion-deletions over the entire non-coding region (999 aligned bases) and therefore represents a distinct haplotype variant of *C. goreaui* (LaJeunesse and Thornhill, 2011). The phylogenetic tree (Supplementary Material 1) based on *psbA*^*ncr*^ regions (1,013 bp in total) show that, GF19C and *Cladocopium goreaui* belong to two closest sister clades, while the entire *Cladocopium* sp. C1 isolates roughly divided into three clades. In sum, the algal strain isolated from *Galaxea fascicularis* and cultured *in vitro* in this study were *Cladocopium* sp. C1 (family: Symbiodiniaceae). We designated this strain as GF19C1.

### Morphology and Ultrastructure of GF19C1

Most of GF19C1 cells were on the coccoid stage of gymnodinioid dinoflagellate, with smaller mean size than their symbiotic congeners (SC). The average long diameter and short diameter of GF19C1 were 9.65 ± 1.07 μm and 8.92 ± 1.18 μm (Table 1), respectively, which were significantly smaller than those of their SC (Table 1, 10.32 ± 0.97 μm and 9.83 ± 0.93 μm, respectively, *P* = 0.003, Kruskal–Wallis test, same below). Contrastingly, the average division ratio of GF19C1 was 17.6 ± 3.7%, significantly higher than that of its SC (9.7 ± 1.7%, *P* = 0.010, Table 1).

**TABLE 1:** Comparison of the morphology and structure of GF19C1 and its symbiotic congeners (SC).

**Morphology/structure**		**GF19C1**	**SC**	**Significance**
Cell size	long diameter	9.65 ± 1.07 μm	10.32 ± 0.97 μm	***P* = 0.003**
	short diameter	8.92 ± 1.18 μm	9.83 ± 0.93 μm	***P* = 0.008**
Cell wall thickness		0.178 ± 0.042 μm	0.061 ± 0.022 μm	***P* = 0.000**
Chloroplast volume density		12.9 ± 3.9%	56.4 ± 0.1%	***P* = 0.000**
Surface density of thylakoid lamellae		10.27 ± 0.91	10.79 ± 1.66	*P* = 0.602
Volume fraction (VF) of vacuole		16.80 ± 8.14%	3.50 ± 1.44%	***P* = 0.009**
VF of accumulation bodies		4.40 ± 2.06%	9.50 ± 6.13%	*P* = 0.347
VF of mitochondria		1.30 ± 0.18%	1.20 ± 0.18%	*P* = 0.456
Cell division ratio		17.6 ± 3.7%	9.7 ± 1.7%	***P* = 0.010**

*P values are obtained from Kruskal–Wallis test. Data are mean ± standard deviation. The significant *P* values are indicated in bold.*

**FIGURE 2:**
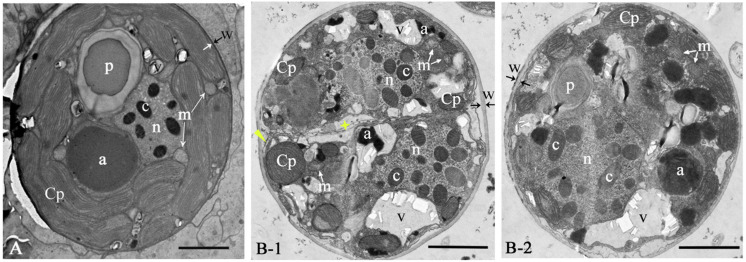
The ultrastructure of *Cladocopium* sp. C1 from coral *G. fascicularis*. **(A)** *in situ*; **(B-1/B-2)** GF19C1, *in vitro* cultured. Panel **(B-1)** is the newly divided cell, showing new cell membranes (star) and cell wall forming (triangle). a, accumulation body; c, chromosome; Cp, chloroplast; m, mitochondrion; n, Nucleus; p, pyrenoid; v, vacuole; w, cell wall, paired arrows indicate the thickness of the cell wall. Scale bars = 2 μm.

The ultrastructure pattern of coccoid stage of GF19C1 (Figure 2) is similar to that of algal symbionts from other coral hosts (Berner and Izhaki, 1994; Wham et al., 2017). The cell walls of GF19C1 (Figure 2B-1/-2) are composed of an electron-translucent material, showing the homogeneous fine granular structure as described by Palincsar et al. (1988) and Lesser and Shick (1990), while the cell wall of their SC (Figure 2A) is dark and thin, lacking the granular layer presented in GF19C1. In addition, the mean thickness of cell walls of GF19C1 is 0.178 ± 0.042 μm, which is significantly thicker than that of its SC (0.061 ± 0.022 μm, *P* = 0.000, Table 1, also see Figures 2A,B-1/-2, coupled arrows).

The symbiont cells of *G. fascicularis* have several well-developed and long-striped chloroplasts that are connected with each other and surround continuously the outer layer of cytoplasm in 1–2 layers (Figure 2A), whereas in GF19C1 cells, the chloroplasts are short and discontinuous (Figures 2B-1/-2). The chloroplast volume fraction of symbiont cells was 56.4 ± 10.1%, which is significantly higher (*P* = 0.000) than that of GF19C1 (12.9 ± 3.9%, Table 1), but there is no discernable difference in the surface density of thylakoid lamellae (SDTL) relative to chloroplast volume (Table 1, 10.27 ± 0.91 vs. 10.79 ± 1.66, *P* = 0.602). The vacuoles are often small and numerous in symbiont cells, with regular shapes of round or ellipse (Figure 2A), while in GF19C1 cells, the vacuole usually presents an irregular large cavity (Figures 2B-1/-2). The vacuole volume fraction of GF19C1 was significantly higher than that of its SC (16.8 ± 8.14% vs. 3.5 ± 1.44%, *P* = 0.009, Table 1). Compared to the symbionts *in hospite*, more freshly divided cells were observed in GF19C1 (Figure 2B-1) and the newly formed cell wall is thin and membranous, instead of vesicular (Figure 2B-1, triangle).

In addition, no significant differences were found in both the volume fractions of mitochondria and accumulation bodies between GF19C1 cells and their SC (Table 1, 1.3 ± 0.18% vs. 1.2 ± 0.18% *P* = 0.456 and 4.4 ± 2.06% vs. 9.5 ± 6.13% *P* = 0.347, respectively).

### Effect of Light and Temperature on the Growth Rate of GF19C1

**FIGURE 3:**
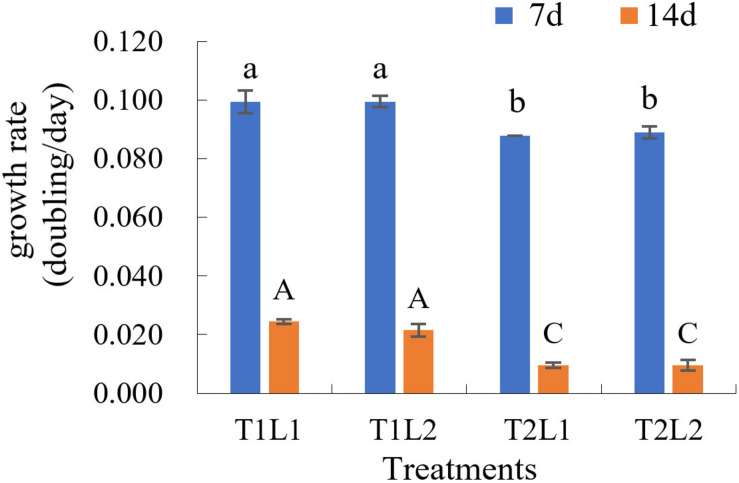
The growth rate (*K*, doubling/day) of GF19C1 in the orthogonal experiments of illumination (weak L1/strong L2) and temperature (low T1/high T2). Each set of treatments has three replicates (*n* = 3). The error bars stand for standard deviation (SD). Superscripts in lowercase and uppercase letters denote twice ANOVA multiple comparisons, followed by Tukey *post hoc* to locate the significant differences. Different letters within the same comparison group mean significant difference (ANOVA, *P* < 0.05).

The results of orthogonal experiments in 8 months post-isolation showed that, at day 7, the growth rates (*K*) of T1L1 (low temperature/weak light) and T1L2 (low temperature/strong light) groups were the highest (both are 0.099, Figure 3 and Supplementary Table 2), which was significantly higher than that of T2L1 (high temperature/weak light) and T2L2 (high temperature/strong light) groups (ANOVA, *P* < 0.05). At day 14, the growth rate of T1L1 and T1L2 were 0.024 and 0.022 (Figure 3 and Supplementary Table 2), respectively, which were also notably higher than that of T2L1 (0.010) and T2L2 (0.010) (*P* < 0.01), indicating that GF19C1 prefers the lower temperature (T1, 25 ± 1 vs. 28 ± 1°C) at which it was consistently maintained ever since the isolation. On the other hand, the impact of light intensities (42 ± 2/62 ± 2 μmol photons m^–2^ s^–1^) in this experiment showed no significant difference on algal growth.

### Effect of Salinity on the Growth Rate of GF19C1

**FIGURE 4:**
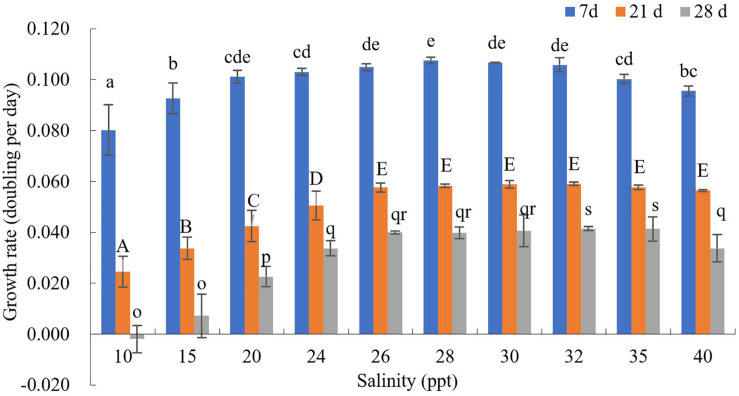
The growth rate (*K*, doubling/day) of GF19C1 under a series of salinity (ppt) gradient. Superscripts in a-e, A-E, and o-s denote three ANOVA multiple comparisons, followed by Tukey *post hoc* to locate the significant differences. Different letters within the same comparison group mean significant difference (ANOVA, *P* < 0.05). Each salinity has three replicates (*n* = 3). The error bars stand for standard deviation (SD).

The effects of different salinity (S) on the growth rate of GF19C1 (12 months post-isolation) were shown in Figure 4 and Supplementary Table 3.

The cell numbers of all salinity groups increased during the first 7-day period. Where the salinity ranging from 10 to 28 ppt, the growth rates increased with the increasing salinities. That is, when *S* = 10, the growth rate (*K*_10_) was the minimum (0.080), while *K*_28_ was the highest (0.108, and the cell density reached to 33.85 × 10^4^ cells/mL) while *K*_30_ is the second (0.107, the cell density was 33.64 × 10^4^ cells/mL). On the contrast, the growth rates decreased with the increase of salinity from 28 to 40 ppt, as *K*_40_ decreased to 0.096. Nonetheless, the variance analysis indicated no significant difference in growth rates when the salinity ranging from 26 to 32 ppt (*P* > 0.05).

At day 21, *K*_30_, and *K*_32_ reached the peak (0.059), where the cell densities were 54.93 × 10^4^ and 55.14 × 10^4^ cells/mL, respectively. Besides, it was observed that the cytochrome gradually faded in the groups of salinity lower than 28 ppt.

At day 28, the cell density declined in all salinity groups. However, the decline in groups *S* ≤ 30 are still higher than that of groups *S* = 32 and 35, which had the highest cell density (50.92 × 10^4^ and 50.71 × 10^4^ cells/mL). Hence, we concluded that the GF19C1 strain can grow normally in the salinity ranging from 26–40 ppt, and the optimum salinity is between 28–32 ppt.

Overall, the growth rate *K* in this test (12 months post-isolation, Figure 4 and Supplementary Table 3) are significantly higher than that of light-temperature orthogonal experiment (8 months post-isolation; Figure 3 and Supplementary Table 2).

## Discussion

The symbiosis between algal symbionts and reef-building corals were thought to emerge in the mid-Triassic period (Stanley, 1981). In the ensuing 230 million years of intense differentiation and speciation of the hermatypic corals (Shepard, 1964), the two mutualistic sides, corals and their endosymbiotic algae, have undergone precise coordination or a series of synergistic mutations and formed obligate interdependence (Antonelli et al., 2016). The photosynthetic symbionts have established relatively stable and complex communities in specific coral species and geographic regions (Lewis et al., 2019), and the symbiont biota also changed with the persistence and periodicity of environmental stress (Lee et al., 2016; Lewis et al., 2019). On the other hand, adapted to the life in coral cells, symbionts and their host have reached a delicate metabolic balance (Lin et al., 2019) and physiological compromises (such as abandoning sexual reproduction and being compatible with the coral’s immune system) (Antonelli et al., 2016). Therefore, when we attempt to isolate the symbionts from coral cells and establish genetically and physiologically consistent strains, the algae should experience drastic morphological and physiological changes so as to adapt to the new artificial culture environment.

### Isolation and *in vitro* Culture of GF19C1

The isolation of algal symbionts from *Galaxea fascicularis* and the establishment of monoclonal strain GF19C1 have undergone a tedious process for more than 1 year, while many efforts have been taken for optimization, including culture medium components, light intensity managements, and contamination control etc. We found that the common medium (e.g., f/2 and L1 medium) could be applied to GF19C1 *in vitro* culture upon minor modification. One of the greatest challenges was to eliminate the contamination of diatoms, protozoa, and fungi in the culture system. Although the use of triple antibiotics (penicillin, streptomycin and kanamycin, final concentration of 200, 100, and 100 μg/mL, respectively, Lin et al., 2015) can effectively inhibit the bacteria growth in the cultures, however, contaminants like fungi, diatoms and protozoa that originated from the coral exoskeleton or internal polyps during isolation, were difficult to eliminate. Chakravarti and Van Oppen (2018) used an antifungal cocktail (consisting of nystatin, amphotericin, and GeO_2_, final concentration 100 μg/mL, 2.5 μg/mL and 50 μM, respectively) to inhibit the contamination of fungi and other organisms during the centrifugal collection of symbiotic algae from plenty of broken tissues. As reported here, we used a different strategy to reduce the sources of contamination. We selected the tentacles of *Galaxea fascicularis*, which were easily sampled and cleaned for symbiont isolation. Meanwhile, we also gently teared down the tissue to avoid the damage of symbiont cells. The algal colonies were then cultured by the soft agar plates containing triple antibiotics. We eventually obtained the stable and passaged algal -strain GF19C1 cell line. The protocol of the established GF19C1 strain mainly followed the monoclonal culture procedure from Schoenberg and Trench (1980a), which is a rather rigorous and tedious approach with the process involving the rejection of the dominant alga to achieve culture purification during the algae culture (Chakravarti and Van Oppen, 2018).

The symbiotic phylotypes associated with *G. fascicularis* were mainly dominated by Symbiodiniaceae ITS2-C1, D1, and C21a, along with numerous of other background clade C phylotypes (Xu, 2019; Wepfer et al., 2020). Even identified as clade C phylotype by n18S-rDNA and *Taq*I-generated RFLPs (Santos et al., 2002), the symbiotic biota in *G. fascicularis* could also possess multiple composition of both dominant and background symbionts when probed with ITS2 sequence tagging. Thus, a more rigorous strategy of isolation and purification process is necessary to achieve the pure cell line.

### Ultrastructural Changes of GF19C1

Our study revealed that the cell wall of *in vitro* cultured GF19C1 could be significantly thicker (about twofold) than that of their symbiotic congeners (SC). This phenomenon is rather common (Lesser and Shick, 1990), but there are several aspects to account for its causes and results. One probable explanation is that, the thinner cell wall of symbionts *in hospite* is an adaptation to live inside coral cells, which may facilitate the transport of nutrients between coral cell and symbiont (Schoenberg and Trench, 1980b). Freudenthal (1962) noted that, the algal symbiont in coral cell is haplontic and autotrophic vegetative cell. When cultured *in vitro*, they become vegetative cyst by thickening the cell wall, which could restore both sexual and asexual reproductive potential through producing autospores, aplanospores, or motile gymnodinioid zoospores, or possible gametes, so that they can adapt to the outside environment (Freudenthal, 1962). Palincsar et al. (1988) also observed the phenomenon of cell wall thickening of algae during growth outside the host sea anemone *Aiptasia pallida*. This was thought to be related to an increase in mitotic rate after isolation, because during the division the entire new cell walls were synthesized resulting thickened newly produced cells (Palincsar et al., 1988). In addition, the mitotic rate of GF19C1 also increased by 8% higher than that of its SC, which was similar to that (10%) of *in vitro* cultured algal symbiont isolated from *A. pallida* (Palincsar et al., 1988).

The volume fraction (VF) of chloroplast of GF19C1 cells was significantly lower than that of their SC, whereas the surface density of thylakoid lamellae (SDTL) showed no obvious change. Unlike their SC, *in vitro* cultured algal symbionts don’t have to provide photosynthetic nutrients for coral cells in exchange for their protection (Davies, 1993). Therefore, their photosynthetic burden is reduced and they don’t need to possess so many photosynthetic apparatuses. Especially when cultured *in vitro*, the light intensity is usually greater than that in host cells. Interestingly, for the algal strain isolated from sea anemone *Aiptasia pallida*, its VF of chloroplast didn’t change but SDTL remarkably decreased, when compared to their SC (Lesser and Shick, 1990). Perhaps, that is another photo-adaptive strategy to a greater outside (*in vitro*) light intensity.

With the notable reduction in chloroplast VF, the vacuoles of GF19C1 cells could join together and merge into one or two larger irregular vacuoles. And the volume density of vacuoles increases significantly, which may favor frequent mitosis in GF19C1. Freudenthal (1962) also found *in vitro* cultured symbiont cells have 1–2 large vacuoles, meanwhile, contain intact chloroplasts and accumulation bodies. Such cells not only do not mean aging, but extensively appear in the culture medium containing actively-reproducing algae. The appearance of these big vacuoles could imply some unknown factors on regulating cell physiology. We speculate that it is likely to be associated with the increased demand for processing metabolic wastes and more flexibility in space as cell division accelerating.

### Suitable Light, Temperature and Salinity Conditions of GF19C1 Growth

Light and temperature are critical environmental factors for algae growth. We found that the initially isolated algal cells show a high sensitivity to light intensity, and might bleach to death in 3 days under 50 μmol photons m^–2^ s^–1^, with 12:12 light/dark cycle. If the light intensity was reduced to 40 μmol photons m^–2^ s^–1^, the survival time of the algal cells could be significantly prolonged. Even within coral cells, a sudden increase in light intensity could also reduce the pigment content rather than decrease the density of symbionts, and result in coral bleaching (Hoegh-Guldberg and Smith, 1989). Generally, light intensity has a greater effect on algal growth than temperature (Sakami, 2000). The appropriate light intensity range for GF19C1 increased from 40 to 64 μmol photons m^–2^ s^–1^ in 8 months post-isolation, when the cell growth rate at 25 ± 1°C was significantly higher (*P* < 0.05) than that at 28 ± 1°C. This may be due to the fact that GF19C1 have been kept consistently at 25 ± 1°C since isolation, which result in an inertia of temperature preference for its growth and division so that they could not adapt to the sudden increase of temperature. The growth rate *K* on 8 months post-isolation was rather low. This may be because, on the one hand, GF19C1 hasn’t adapted to the extracellular environment yet, or on the other hand, the restrained influence of host cytokines is still present (Cunning et al., 2015). When GF19C1 was cultured for 12 months *in vitro*, the growth rate *K* increased to 0.108, and showed more rapid evolution than the coral host (Chakravarti and Van Oppen, 2018). Although this *K* value was still low compared with other free-living dinoflagellates (Liang et al., 2011), it is comparable to that of other algal symbionts cultured *in vitro* (Chakravarti and Van Oppen, 2018).

Given that when GF19C1 were transferred from coral cells to the external environment, they may be more sensitive to salinity changes. Therefore, a dense gradient (ten gradients. from 10 to 40 ppt) was set to determine the optimum salinity *in vitro*. The effect of salinity on aquatic organisms is mainly manifested in the regulation of osmotic pressure in cells (Sakami, 2000). At day 7, the density of GF19C1 increased in all salinity groups (10–40 ppt, Supplementary Table 3). As the experiment went on, comparing with the high salinity groups, the growth of low salinity groups (10–24 ppt) decreased more significantly (Figure 4 and Supplementary Table 3) because of the accumulation of osmotic pressure (Maboloc et al., 2015), at day 21, we can observe that the cytochrome gradually faded in the low salinity groups. Compared with the algal symbionts on mantle of juvenile giant clam *Tridacna gigas*, which display acclimation response to salinity of 25 ppt (Maboloc et al., 2015), GF19C1 was more sensitive to low salinity stress. In addition, we also observed that, the cell density declined in all salinity groups at 28 days post-experiment, that is because the batch culture model was applied during the test and at that time the algae was at the Death/Lysis phase (Farag and Price, 2013).

GF19C1 cells can grow normally in a salinity range of 26–40 ppt, and its optimum salinity is 28–32 ppt, comparable to the suitable salinity (32–40 ppt) of most corals (Veron, 1986). In suitable ranges, the effects of salinity on algal growth are not as obvious as that of light and temperature (Hoegh-Guldberg and Smith, 1989).

To sum up, GF19C1 is the first *in vitro* cultivated monoclonal strain isolated from the endosymbiotic biota of the scleractinian coral *Galaxea fascicularis*. When cultured *in vitro*, GF19C1 cells show morphological changes, preparation for the recovery of sexual reproduction, rapid adaptation to light intensity, and rapid evolution of growth rate. Symbiodiniaceae C1 are the most widespread algal symbiont in reef-building corals (Stat et al., 2008), their distribution ranged from high latitude region e.g., Korea-Jeju Island and Japan, to tropical region e.g., South China Sea (Reimer et al., 2006; Ng and Ang, 2016; Chen et al., 2019, 2020; Wepfer et al., 2020), thus they have experienced large sea surface temperature variations and high turbidity (Ng and Ang, 2016; Wepfer et al., 2020). Therefore, in future study it is necessary to further explore the adaptive potential of GF10C1 to multiple environmental factors and high gradient changes. The establishment of monoclonal culture technology will make it possible for the isolation and *in vitro* culture of *G. fascicularis* and other hermatypic corals-associated algal symbiont species, which is important to elucidate the physiological and ecological characteristics of various species of symbiont, and their cooperative mechanisms within coral hosts, and will help to further clarify their role and function in the environment adaptation of scleractinian corals.

## Data Availability Statement

The original contributions presented in the study are included in the article/Supplementary Material, further inquiries can be directed to the corresponding author/s.

## Author Contributions

JW, JC, and SW contributed equally to this study. YW was responsible for conceptualization, funding acquisition, and resources. JW, JC, FL, CF, and SW were responsible for experimental investigation. JW and JC were mainly responsible for data processing and picture modification. YW, JW, and JC were responsible for the draft preparation. YW proofread the final manuscript before submission. All authors have read and approved the final manuscript.

## Conflict of Interest

The authors declare that the research was conducted in the absence of any commercial or financial relationships that could be construed as a potential conflict of interest.
