# Supplementary material for: Monoclonal Culture and Characterization of Symbiodiniaceae C1 Strain From the Scleractinian Coral Galaxea fascicularis
Source: Front Physiol. 2021 Jan 18;11:621111. doi: 10.3389/fphys.2020.621111 (PMC7848188; doi:10.3389/fphys.2020.621111)

**Sequences of ITS1 ITS2, psbA and phylogeny trees of GF19C1**

1. The followings are the sequencing (bidirectional direct Sanger sequencing and splicing, the primers of the splicing sequences were marked) results of ITS1 and ITS2 of GF19C1. The ITS1 amplicon is 334 bp, and the ITS2 amplicon is 287 bp, excluding the primer’s sequences (marked in green).

ITS1 sequence:

>symITS1_GF19C1

TTTTGCTGCGCCCTTCACAGGTGCCCGAGCCAAGATATCCATCGCTGAAAGTTGTGGAAGTTGGAACAATGCCCTTCCCAGACAGTTGTCACTAAAGCAGCTTAGCCTTCATTCCCCGGCCCGCTGAACACGGACCCATGGCCACAGAGCGACACTCGCACCAGCCCCAACAAGGGTTGGTGAGTGTGCATCTGTCGCCCTCGAGTTCTGCAAGCAGATATCTCACCGCAAGCATCCCTCACAGCCAAAATTCACGTAGTCAAAATTGAATCAGTGCGAATGATCCTTCCGCAGGTTCACCTACGGAAACCTTGTTACGACTTCTCCTTCCTCTAAGTGATAAGGTTCATGAAACTTTCCAACGCAACGTCAAGAAGCTGAGAAATTTCCTAAGGCTG

ITS2 sequence:

>symITS2_GF19C1

AACTCCGTGAACCAATGGCCTCCTGAACGTGCGTTGCACTCTTGGGATTTCCTGAGAGTATGTCTGCTTCAGTGCTTAACTTGCCCCAACTTTGCAAGCAGGATGTGTTTCTGCCTTGCGTTCTTATGAGCTATTGCCCTCTGAGCCAATGGCTTGTTAATTGCTTGGTTCTTGCAAAATGCTTTGCGCGCTGTTATTCAGGTTTCTACCTTCGTGGTTTTACTTGAGTGACGCTGCTCATGCTTGCAACCGCTGGGATGCAGGTGCATGCCTCTAGCATGAAGTCAGACAAGTGAACCCGCTGAACTTAAGCAATATGGATCCAACCCTCCCCCTTTTAT

2. *psbA^ncr^* sequence of GF19C1

>psbA_GF19C1

TGATGTGGTTAGGGGTACCCTAATATTGGCCTGTTTTAGGTGCCTCTATGGGTACTTCTATGGGTACCAGCCTAATAATTTTGACATCAACCCTTTGATTTAGGGTACCCAGCATAGTACCCATAAGGGGTGCCCTATTCCCCACACCCAAACAGGTACCCTAATTTAGACCAGAATTTAGGCCACATCATAAATTAGTGAGTTTTTTCACCCCGAAGGGATGGGTGCCCCTTGTGGGTACCCATATGCCCGCAGGGCATTGAGGTCCACGAAGTGTGACCGTTAATTTTGGCCAAAAAAAGGGTGCCGCTAATTTTGGCCTAAACTGCGACTATTTTGGCCAAAATTGCGACATGTGTCCTCGCGCACACGCGCACACGTATTAAAAAGATCGCGACTTATCGTGACGTAAATGGGTGCCCCTGTGGGGCACCCATATGCACCACCCTTTGGGTGGTGCATTGGAGCCGGGAACGAGCCGAAGGCGAGTGGACGTGCGACCACGAAGAAAAGAAAAAGAAATCGCGACCTATAATGGGTGCCCCTTTGGGGCACCCATATGCACACCCCGAAGGGGTGTGCATTAATGGGCTGGGTGCCCTACCCAGCCCATATGGGCCCACGCTTCGCGGGGCCCATAACGGCCCTTCGGGCCTTCAAAAATAAAAAAGTAATGATGGGTGCCAGTAACCGAGCAAAATAACCAAGCAAATCAACCTGCAGGCAATAATGGCCAAAACCTGTGCGAACCAAGTGTGAATCTGCGGCTATGTCTCGGGGTGTGCATATGCCTGCTGGCTGCCTGCACATGTGCCCTCCACCCCTACCCCTCCTCCCAACAGAAGAATCTGGCCAAAATTATCATCACAATCTTTTGCAGGTGATTGATTACTACCAATGAAGAACACATCTTACTATCAACTCAACTTACTTGGTAATGTCATAGGATTCGTGCTATCCACAACAAATCGTCTCTACATTGGGTGCTTTGGTATCCTAATGTTCCCTCTTTT

3. NJ Phylogeny tree based on ITS1 sequences:


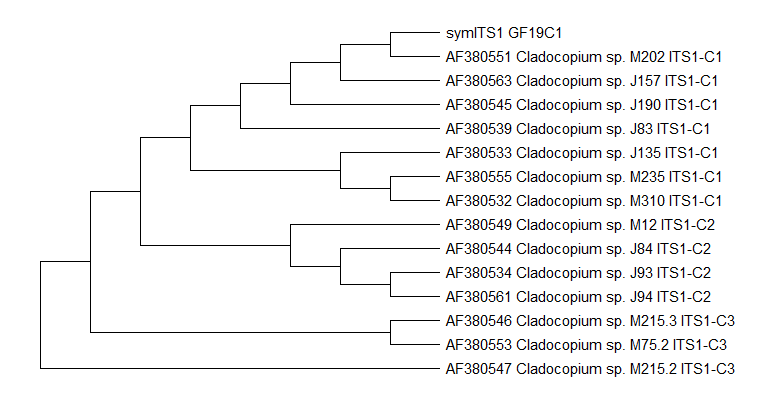


4. NJ Phylogeny tree based on ITS2 sequences:


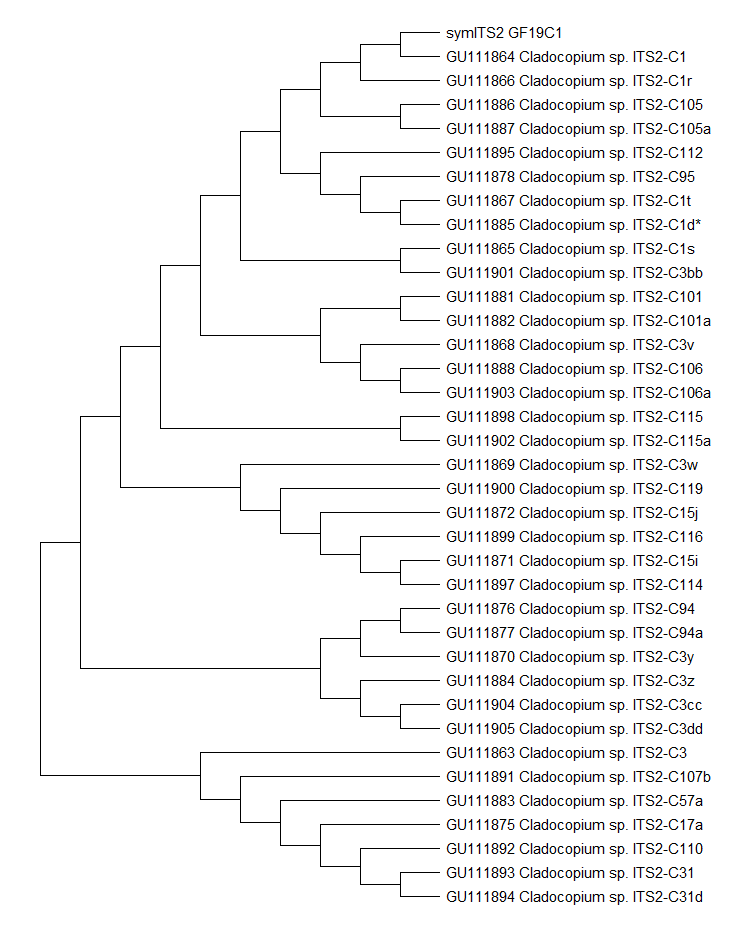


5. NJ Phylogeny tree based on psbA sequences:


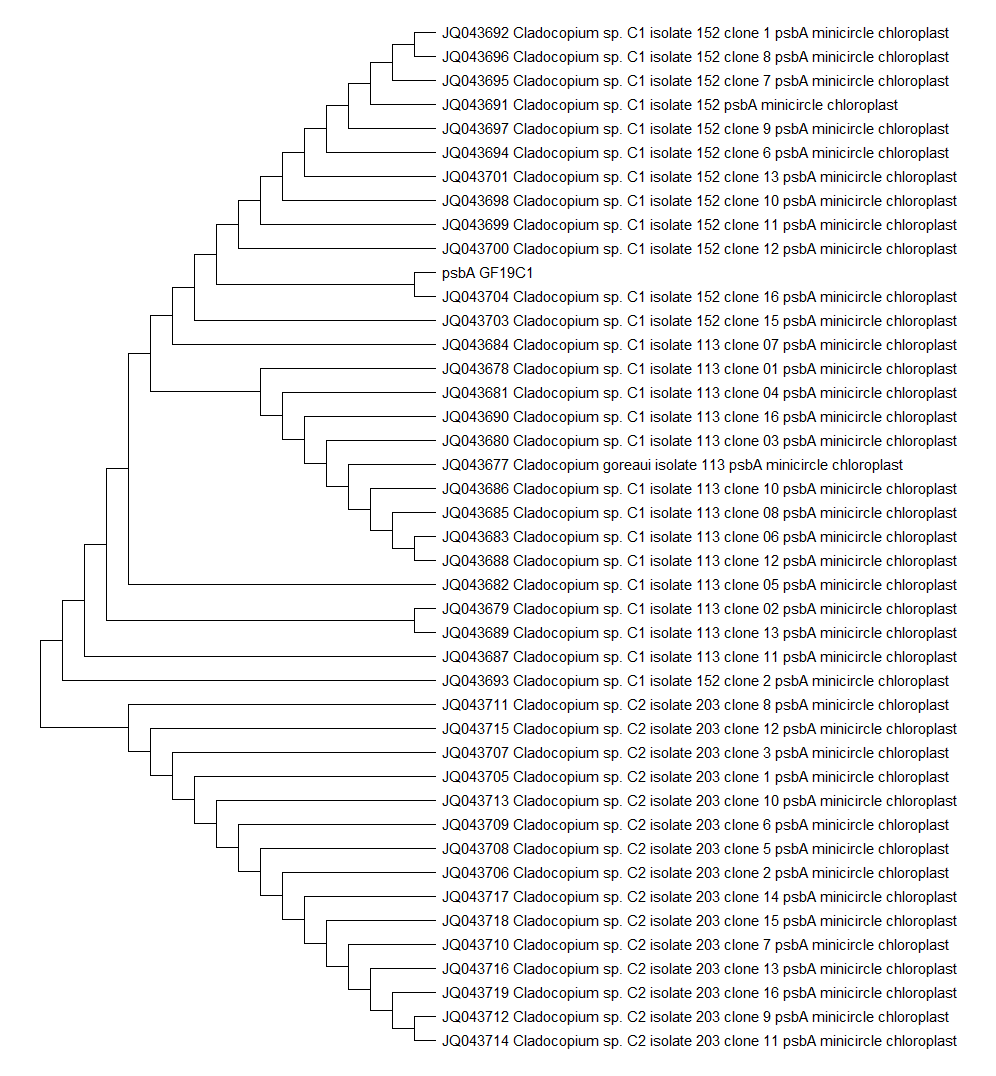

Supplement: Supplementary file 1 [file Data_Sheet_1.DOCX]
