# Supplementary material for: Monoclonal Culture and Characterization of Symbiodiniaceae C1 Strain From the Scleractinian Coral Galaxea fascicularis
Source: Front Physiol. 2021 Jan 18;11:621111. doi: 10.3389/fphys.2020.621111 (PMC7848188; doi:10.3389/fphys.2020.621111)
Supplement: Supplementary file 2 [file Data_Sheet_2.docx]

**Supplementary Figure and Tables:**


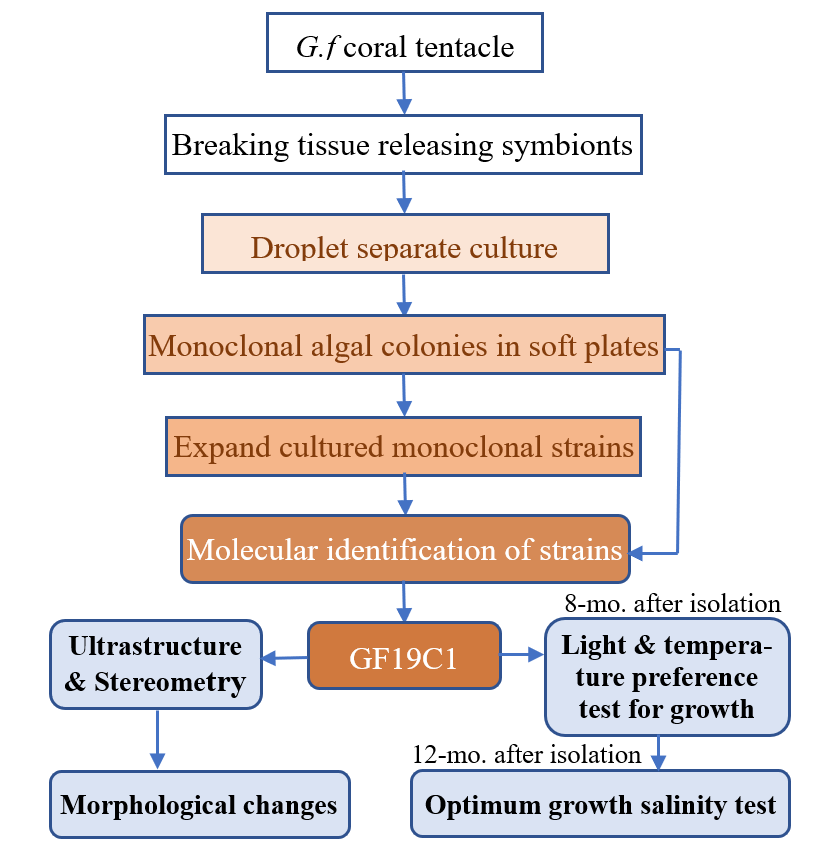


**Figure S1** Experimental design.


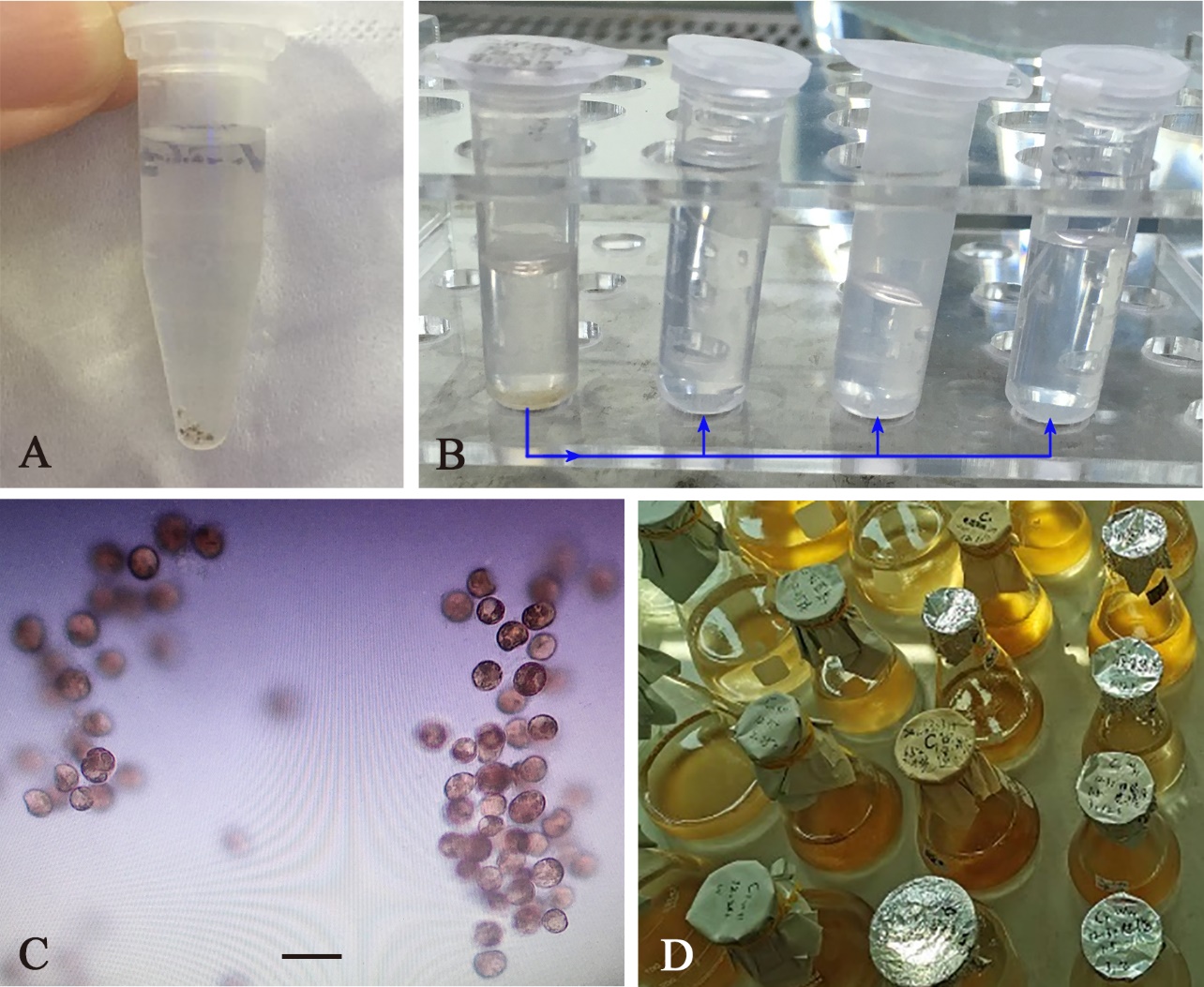


1

2

3

4

**Figure S2** Isolation, purification and *in vitro* culture of GF19C1

(A) The perioral tentacles were sampled, and were rinse with sterilized sea water (the sediments are the broken septum or other adhesive pieces); (B) By blowing and sucking with a pipette tip, the algal symbionts were released and collected in tube 1, by droplet method, the algal cells were separated to tube 2, 3 and 4, and were cultured with Z1 medium; (C) The separated algal cells were spread on the plate, about 10-12 weeks later, monoclonal algal colonies were observed, scale bar = 20 μm; (D) The monoclonal alga was transferred to Z1 medium for expand culture.

**Table S1** The component of Z1 Medium

| NaNO_3_ | 75 mg/L |
| --- | --- |
| NaH_2_PO_4_.H_2_O | 5.0 mg/L |
| Na_2_SiO_3_.9H_2_O | 20 mg/L |
| FeC_6_H_5_O_7_.5H_2_O | 3.9 mg/L |
| Vitamin B_12_ | 0.5 μg/L |
| Biotin | 0.5 μg/L |
| Vitamin B_1_ | 100 μg/L |
| ZnSO_4_.4H_2_O | 20 μg/L |
| MnCl_2_.4H_2_O | 175 μg/L |
| CuSO_4_.5H_2_O | 10 μg/L |
| NaMoO_4_.2H_2_O | 7.0 μg/L |
| CoCl_2_.6H_2_O | 12 μg/L |
| Na_2_EDTA | 4300 μg/L |

**Table S2** The effect of temperature and illumination on the growth rate of C1 zooxanthellae *in vitro*.

| Groups | Initial density  (10^4^ cells/mL) | 7 d density | 14 d density |
| --- | --- | --- | --- |
| T1L1 | 13.41 | 26.90±0.73^a^ | 37.86±0.37^A^ |
| T1L2 | 13.41 | 26.90±0.37^a^ | 36.38±0.97^A^ |
| T2L1 | 13.41 | 24.79±0.00^b^ | 28.37±0.97^C^ |
| T2L2 | 13.41 | 25.00±0.37^b^ | 28.59±1.26^C^ |

| Groups | 7 d growth rate | 14 d growth rate |
| --- | --- | --- |
| T1L1 | 0.099±0.004^a^ | 0.024±0.001^A^ |
| T1L2 | 0.099±0.002^a^ | 0.022±0.002^A^ |
| T2L1 | 0.088±0.000^b^ | 0.010±0.002^C^ |
| T2L2 | 0.089±0.002^b^ | 0.010±0.003^C^ |

Note: The same superscript letters represent no significant difference between cell densities of growth rates (*P*>0.05), connected superscript letters represent significant differences (*P*<0.05).

**Table S3** The effect of salinity on the growth rate of C1 zooxanthellae *in vitro*.

| Salinity | Initial density  (10^4^ cells/mL) | 7 d density | 21 d density | 28 d density |
| --- | --- | --- | --- | --- |
| 10 | 15.94 | 27.95±2.53^a^ | 26.69±3.52^A^ | 15.10±1.93^o^ |
| 15 | 15.94 | 30.90±1.59^b^ | 32.38±2.90^B^ | 19.10±5.74^o^ |
| 20 | 15.94 | 32.38±0.63^cde^ | 38.91±4.83^C^ | 30.27±4.21^p^ |
| 24 | 15.94 | 32.80±0.37^cd^ | 46.29±5.51^D^ | 41.02±3.24^q^ |
| 26 | 15.94 | 33.22±0.37^de^ | 53.45±3.18^E^ | 48.81±0.00^qr^ |
| 28 | 15.94 | 33.85±0.37^e^ | 54.29±0.97^E^ | 48.60±2.56^qr^ |
| 30 | 15.94 | 33.64±0.00^de^ | 54.93±1.93^E^ | 49.87±7.65^qr^ |
| 32 | 15.94 | 33.43±0.97^de^ | 55.35±0.37^E^ | 50.92±0.73^s^ |
| 35 | 15.94 | 32.17±0.37^cd^ | 53.66±0.97^E^ | 50.71±6.66^s^ |
| 40 | 15.94 | 31.11±0.63^bc^ | 52.19±0.37^E^ | 41.23±6.10^q^ |

| Salinity | 7 d growth rate | 21 d growth rate | 28 d growth rate |
| --- | --- | --- | --- |
| 10 | 0.080±0.013^a^ | 0.025±0.007^A^ | -0.002±0.005^o^ |
| 15 | 0.093±0.007^b^ | 0.034±0.004^B^ | 0.007±0.010^o^ |
| 20 | 0.101±0.003^cde^ | 0.042±0.006^C^ | 0.023±0.005^p^ |
| 24 | 0.103±0.002^cd^ | 0.051±0.006^D^ | 0.034±0.003^q^ |
| 26 | 0.105±0.002^de^ | 0.058±0.003^E^ | 0.040±0.000^qr^ |
| 28 | 0.108±0.002^e^ | 0.058±0.001^E^ | 0.040±0.002^qr^ |
| 30 | 0.107±0.000^de^ | 0.059±0.002^E^ | 0.041±0.006^qr^ |
| 32 | 0.106±0.004^de^ | 0.059±0.000^E^ | 0.041±0.001^s^ |
| 35 | 0.100±0.002^cd^ | 0.058±0.001^E^ | 0.041±0.005^s^ |
| 40 | 0.096±0.003^bc^ | 0.056±0.000^E^ | 0.034±0.006^q^ |

Note: The same superscript letters represent no significant difference between cell densities of growth rates (*P*>0.05), connected superscript letters represent significant differences (*P*<0.05), interval superscript letters represent extremer significant differences (*P*<0.01).
